# Partial Bone Formation in Additive Manufactured Porous Implants Reduces Predicted Stress and Danger of Fatigue Failure

**DOI:** 10.1007/s10439-019-02369-z

**Published:** 2019-09-23

**Authors:** Vee San Cheong, Paul Fromme, Melanie J. Coathup, Aadil Mumith, Gordon W. Blunn

**Affiliations:** 1 John Scales Centre for Biomedical Engineering, Institute of Orthopaedics and Musculoskeletal Science, Royal National Orthopaedics Hospital, University College London, Stanmore, HA7 4LP UK; 2 Department of Mechanical Engineering, University College London, London, WC1E 7JE UK; 3 College of Medicine, University of Central Florida, Orlando, FL 32827-08 USA; 4 School of Pharmacy and Biomedical Sciences, University of Portsmouth, Portsmouth, PO1 2DT UK

**Keywords:** Stress shielding, Rehabilitation, Bone remodelling, Laser sintered titanium alloy implant, Fatigue, Finite element analysis

## Abstract

New porous implant designs made possible by additive manufacturing allow for increased osseointegration, potentially improving implant performance and longevity for patients that require massive bone implants. The aim of this study was to evaluate how implantation and the strain distribution in the implant affect the pattern of bone ingrowth and how changes in tissue density within the pores alter the stresses in implants. The hypothesis was that porous metal implants are susceptible to fatigue failure, and that this reduces as osteointegration occurs. A phenomenological, finite element analysis (FEA) bone remodelling model was used to predict partial bone formation for two porous (pore sizes of 700 μm and 1500 μm), laser sintered Ti_6_Al_4_V implants in an ovine condylar defect model, and was compared and verified against *in vivo*, histology results. The FEA models predicted partial bone formation within the porous implants, but over-estimated the amount of bone-surface area compared to histology results. The stress and strain in the implant and adjacent tissues were assessed before, during bone remodelling, and at equilibrium. Results showed that partial bone formation improves the stress distribution locally by reducing stress concentrations for both pore sizes, by at least 20%. This improves the long-term fatigue resistance for the larger pore implant, as excessively high stress is reduced to safer levels (86% of fatigue strength) as bone forms. The stress distribution only changed slightly in regions without bone growth. As the extent of bone formation into extensively porous bone implants depends on the level of stress shielding, the design of the implant and stiffness have significant influence on bone integration and need to be considered carefully to ensure the safety of implants with substantial porous regions. To our knowledge this is the first time that the effect of bone formation on stress distribution within a porous implant has been described and characterised.

## Introduction

**Figure 1:**
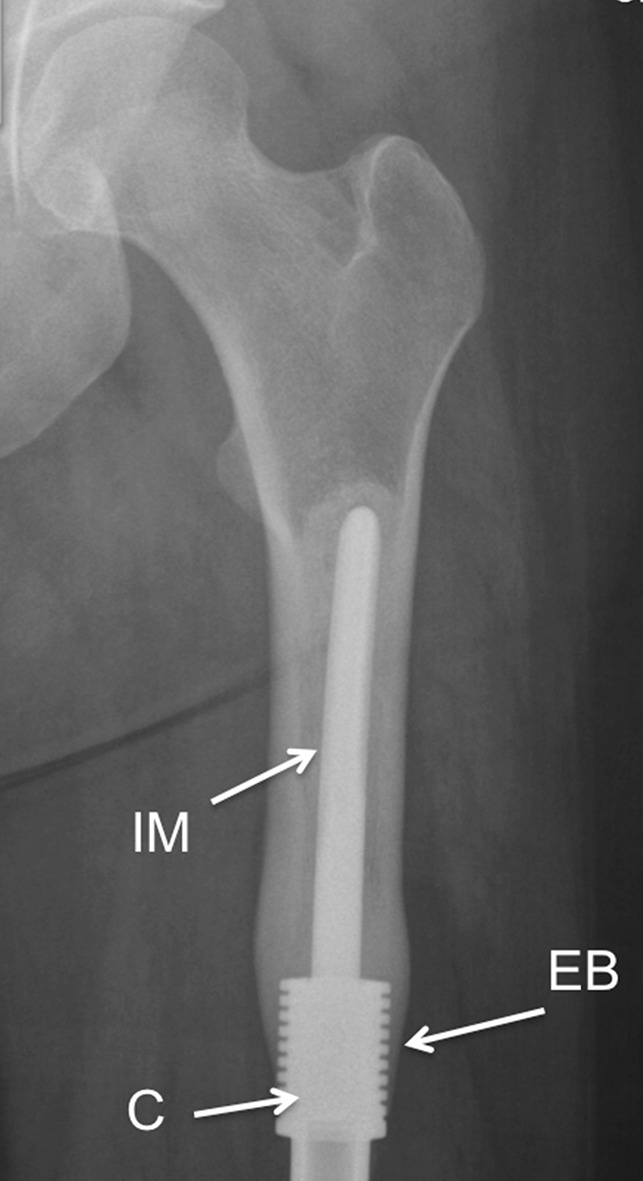
Distal femoral implant 12 years after insertion, showing grooved HA coated collar (C), extracortical bone formation (EB) and cemented intramedullary stem (IM).

The introduction of an implant changes the mechanical environment, resulting in bone adaptation. Bone resorption and aseptic loosening caused by stress shielding can reduce the implant longevity.^11^,^29^,^31^ Retrospective clinical follow-up for bone cancer survivors demonstrated that promoting extracortical bone formation and osseointegration at the shoulder of distal femoral segmental prostheses using a grooved collar design is effective at reducing failure due to aseptic loosening and improves the survivorship of implants at 10 years from 75% to 98% 9 (Fig. 1). An FEA study to understand the effects of extracortical bone formation on stresses in the intramedullary stem found an 80% decrease in stress concentration due to bone growth, protecting the implant from failure at the stem-collar interface.^12^

Recent advancements in additive manufacturing enable implant scaffold designs with extensive interface connectivity to enhance bone growth into porous structures, improving stability and fixation.^18^,^30^,^33^ An *in vivo* study compared the outcomes of selective laser sintered (SLS) porous and machined grooved collars in segmental prostheses and found that porous designs had higher osseointegration.^22^ An experimental study found that filling extensively porous implants with epoxy improved their mechanical properties by a factor of 2-7,^14^ demonstrating the importance of bone formation in additively manufactured porous implants. However, the long-term performance and potential risk of failure of additively manufactured implants is a concern with radiographic evidence of poor osseointegration in short- to mid-term follow-up,^6^,^36^ and *in vivo* evaluation of osseointegration is unable to evaluate safety performance as the test duration is short compared to the implant lifespan in humans. Specified standard mechanical tests are used to determine the fatigue performance of implants, however these tests fail to mimic the different patterns of bone ingrowth and integration.^14^,^35^ Bone formation in the porous structure reinforces the implant and enhances its performance,^3^,^26^ but incomplete bone formation could cause failure due to the partially fused, relatively weak porous material.

Computer simulations are useful for modelling adaptive changes caused by implant architecture,^11^,^33^ to assess the effects on stress and strain within the bone and implant,^12^,^14^ and to compare with the fatigue strength. FEA assessment of changes in the mechanical environment often assumes full bone formation with homogenous material properties.^12^,^14^ However, scanning electron microscopy (SEM) and histology results from animal studies have shown that bone formation and tissue regeneration occur only partially and with varying bone density.^2^,^10^,^20^,^35^ The assessment of long-term performance in terms of bone ingrowth and its effects on the structural integrity is important for porous metal implants as the structures have thin walls and irregular surfaces that can affect fatigue properties, particularly in notch sensitive materials such as titanium alloy.^15^,^21^ One method of modelling adaptive bone changes is through mechanotransduction algorithms, using mechanobiological or phenomenological approaches.^11^ The former is focussed on the short term, modelling the process by which mesenchymal stem cells (MSCs) differentiate to osteocytes and form bone.^4^,^5^ The latter is based on the idea of mechanostat, and is concerned with long-term effects at the tissue level.^16^,^17^ Advantages and limitations of both modelling approaches for orthopaedic implants have been compared.^29^ Mechanobiological models have predicted bone ingrowth on coated surfaces and porous implants,^4^,^5^,^19^,^25^ but as these scaffolds are non-metallic, fatigue behaviour is usually not evaluated. For metallic implants, bone remodelling algorithms to simulate long-term bone density changes are used to conduct stress analysis. Bone remodelling algorithms have also been coupled with a placeholder and an osteoconnectivity matrix to predict the extent of partial, inhomogeneous bone formation in a grooved titanium segmental prosthesis in a verified adaptive FEA model.^7^

The aim of this study was to evaluate the effect of partial bone formation in two porous, laser sintered Ti_6_Al_4_V implants on the internal stress and strain, and the potential implications for implant safety and failure. This paper uses the dataset from a FEA model that was verified with histology results for predicting bone formation into porous implants.^8^ Its focus was the effect of pore size, coating, and material properties on bone formation,^8^ whilst the results reported here investigate the impact of bone formation on the implant. Stress changes due to inhomogeneous tissue densification in the bone and implant were investigated. As histology results were obtained only at one time point, the process of bone ingrowth, where tissue differentiates to bone and becomes rigidly fixed to the implant,^4^,^5^,^19^ was not considered. The changes in tissue density and stiffness with time were modelled using bone remodelling algorithms,^7^,^8^,^17^ considering only the contribution to load carrying capacity from lamellar bone that had formed, but not the smaller effect of immature soft tissue. Stress and strain in the implant and tissues before and after bone remodelling were compared, in relation to the strength and fatigue limit of the materials.

## Materials and Methods

### Animal Model

**Figure 2:**
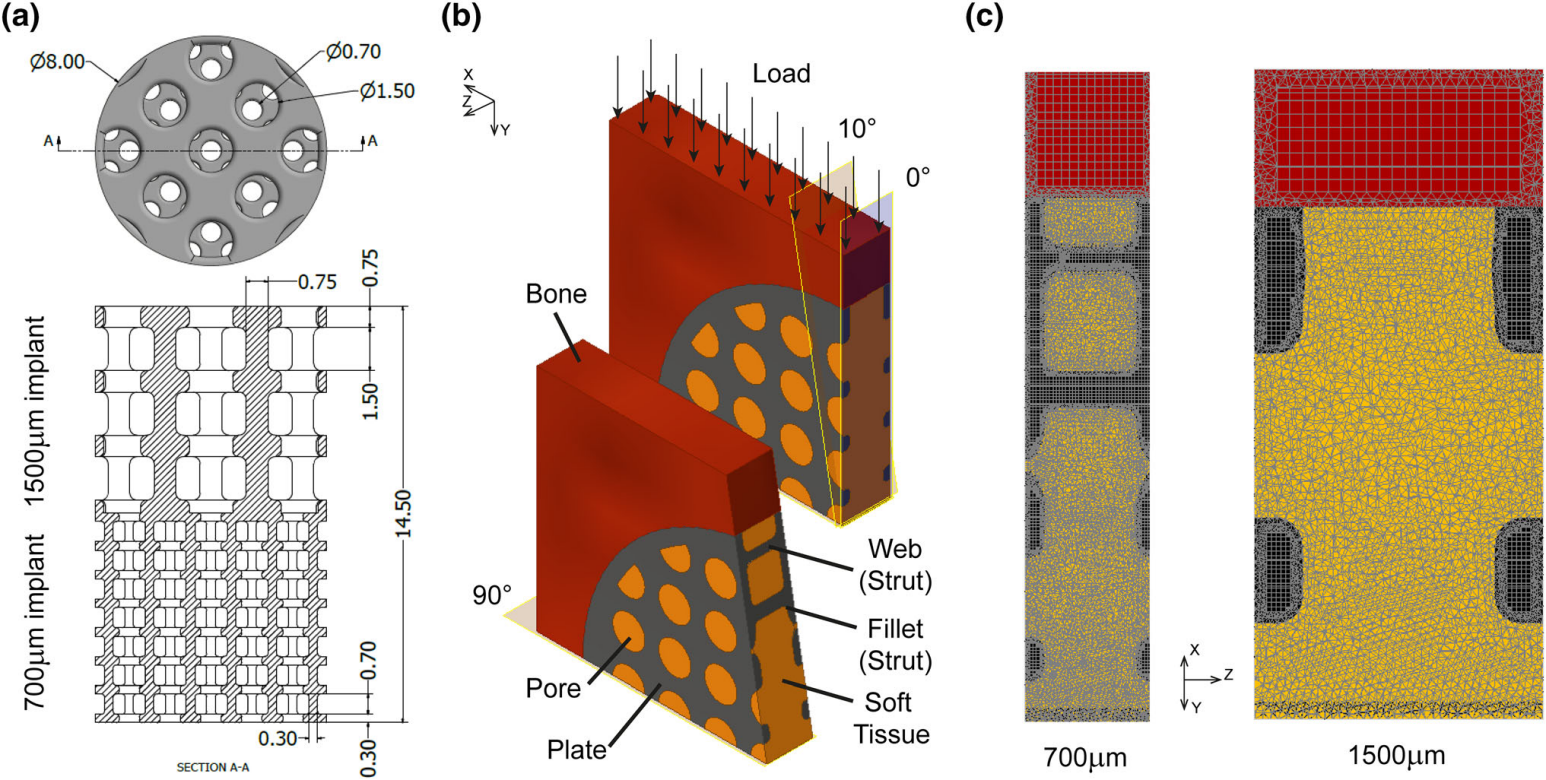
(**a**) Implant design used for *in vivo* sheep study (units: mm); (**b**) FEA model for 700 μm pore size implant; (**c**) mesh at 10° cut from loading direction for 700 μm and 1500 μm implants.

**Figure 3:**
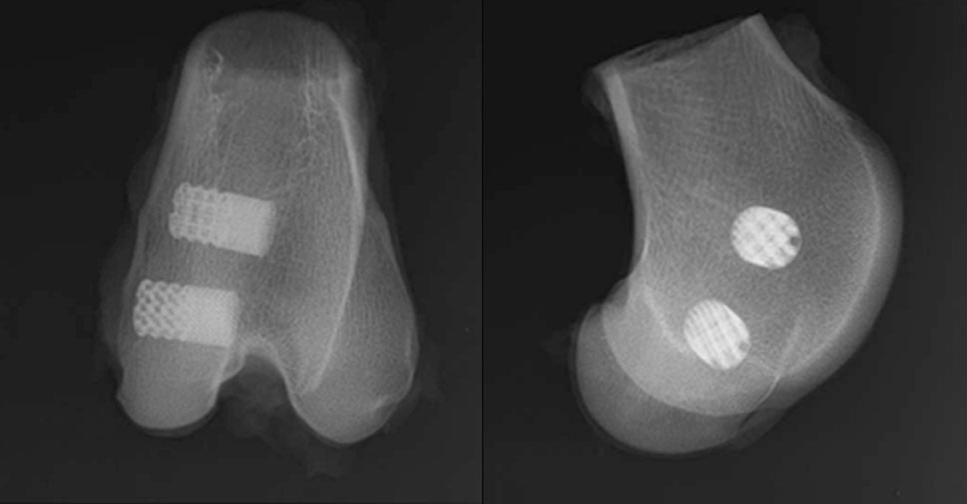
Radiograph showing implant and defect positions within the femoral condyle.

Porous implants (8 mm diameter, 14.5 mm length) with pore sizes of 700 μm and 1500 μm (Fig. 2A) were used as part of a larger study that evaluated the effect of the combination of pore size and different types of HA coatings on osseointegration.^8^ The implant design utilised a strut and plate structure, in a critical sized defect model. The 700 μm implant had struts 300 μm in diameter and 700 μm tall, while the 1500 μm implant had struts 750 μm in diameter and 1500 μm tall. The thickness of the plates corresponded to the diameter of the struts. The 700 μm and 1500 μm implants had porosities of 75% and 70% respectively. The two designs were manufactured as one cylindrical implant using SLS of Ti_6_Al_4_V. Bilateral 8 mm × 15 mm defects were created in the medial femoral condyles of the hind limbs of 6 mature sheep, and the implants pushed into place to achieve a line-to-line fit in the cancellous bone 8 (Fig. 3). The orientation and location (distal–proximal, left and right leg) of the implant was randomized. 30 cylinders were used (24 coated with calcium phosphate), but only results from the 6 uncoated implants (control group) were considered here as the FEA model cannot accurately predict differences between the different types of HA coating. As the uncoated implant had the least amount of bone formation, it represents the worst-case for failure analysis. Tissue and skin were closed, and all animals recovered well. Due to welfare considerations associated with the reduction of animals in experiments, only one time period was used. All procedures were conducted in accordance with the UK Animals (Scientific Procedures) Act 1986, under personal and project licences from the UK Home Office following review by the local animal welfare and ethical research committee.

After 6 weeks, the implants and surrounding bone were retrieved and fixed in formalin. The specimens were dehydrated and embedded in acrylic resin. Longitudinal thin sections approximately 80 μm thick were prepared by sectioning each specimen through the centre, using grinding and polishing techniques. Toluidine Blue and Paragon staining were used to identify soft tissue and bone within the implant, stained purple and red respectively. Imaging of the stained slides was performed using light microscopy (Axioskop, Carl Zeiss, UK). Thresholding and freeform tools in ImageJ (v1.51, NIH, USA) were used to outline bone regions and quantify bone area ratio. Back scattered scanning electron microscopy (SEM) was used to examine bone structure (JEOL 3500C, Japan). Mann–Whitney U tests were conducted in Origin 2016 (OriginLab Corp., USA) to compare bone ingrowth.

### Finite Element Model

Separate FEA models were developed for the 2 pore sizes (Fig. 2), modelling quarter slices using symmetric boundary conditions and repeating pattern to minimise computational costs as the implant orientation and location were not found to affect the results significantly.^8^ Both models had dimensions of 5 mm x 5 mm. Soft tissue was assumed to initially fill the implant pores. Homogenised trabecular bone was assumed to surround the exterior of the implant, as limited geometrical information on the trabecular bone was available. The models were axially restrained. The peak load through the medial condyles of the stifle joint during walking was applied.^28^ This is equivalent to loads of 89 N and 200 N in the 700 μm (1 mm width) and 1500 μm (2.25 mm width) models respectively, scaled according to the repeating pore and strut pattern width. Static loading with specified peak load has been demonstrated to capture the main changes in bone remodelling, comparable to the daily load history of the Stanford model.^16^ Meshes were generated in MSC.Marc 2017.0 (MSC Software Corporation, USA) using linear tetrahedral elements for soft tissue and linear hybrid 4-, 6- and 8-noded elements for bone and implant. This improved geometrical conformance while minimising the total number of elements. Linear elements were used for the soft tissues as mainly compressive load was transmitted through the condyles. The element size was small to capture the geometry around the implants, and the adaptive models were computationally expensive to run. Micromotion was assumed to be minimal and thus tied boundary conditions were used for all contact interfaces. Static stress analysis was conducted to investigate mesh convergence with criteria of 5% error at the fillet and 1% in the soft tissue and bone. The FEA models converged with edge lengths of 0.06 mm and 0.03 mm, yielding 1.33 million and 2.77 million elements for the 1500 μm and 700 μm implants respectively.

Isotropic homogenous material properties were assigned for trabecular bone and implant (Bone: elastic modulus *E* = 1.5 GPa, Poisson’s ratio *ν *= 0.34; Ti_6_Al_4_V: *E* = 110 GPa, *ν *= 0.34).^23^ Soft tissue was assigned an initial modulus *E* = 0.5 GPa, and Poisson’s ratio *ν *= 0.3. Based on the adaptive elasticity theory, tissue density and thus elastic modulus were permitted to adapt according to the strain energy density per unit bone mass (SED) to model bone formation.^7^,^11^,^17^ The model enforces the sequential laying down of new bone by allowing only elements adjacent to bone, and with SED above the threshold, to adapt their density. Elements that did not fulfil these two conditions had no change in density. The physiological basis is that MSCs diffuse from the bone stock to the stimulus site to form new bone,^11^,^16^ as bone formation occurs from the edge of the implant towards the centre of the porous structure. In this model no bone formation occurred spontaneously within the centre of the implant.^7^ Both bone and soft tissue could exist at the initial modulus of 0.5GPa. The conditions imposed led to initial increases in modulus during ingrowth (*E*_i_ = 0.5 GPa), with density decreases due to redistribution occurring only later (number of active elements constant). Change in density *ρ* was calculated from SED:

1 $$\frac{{{\text{d}}\rho }}{{{\text{d}}t}} = B\left( {{\text{SED}} - k} \right) 0 < \rho \le \rho_{\text{cb}}$$

where *B* and *k* are remodelling rate and reference threshold, set at values of 1 (g cm^−3^)^2^/MPa-ctu and 0.0044 J g^−1^ respectively. The upper limit for bone formation *ρ*_cb_, corresponded to 12 GPa.^17^,^31^ Density of surrounding trabecular bone was assumed to remain unchanged, as resorption of existing bone stock due to implant stress shielding is observed only during long-term follow-up.^9^

The stiffness matrix was updated in each time increment using an established density-modulus relationship from literature (*E* = 3790*ρ*^3^).^17^ Density-modulus relationships calibrated for CT images 1 were not employed, as initially homogenous material properties were assigned. Fixed time step of 0.1 computer time unit (ctu) was used until the number of remodelling elements remained stable. For computational efficiency, adaptive time stepping was used thereafter until equilibrium, at 1.2 × of the previous time step.^7^,^8^ Equilibrium was achieved when the change in average tissue density was less than 0.005%. Parametric analysis conducted previously showed that the choice of initial modulus did not affect equilibrium results, but that the initial time step has to be sufficiently small to prevent a numerical overshoot.^7^ Time units used should be considered arbitrary, as the histology results were only available at 6 weeks. There is insufficient literature for correlating ovine bone formation between simulation (ctu) and actual time, and the time correlation found previously for human subjects may not be applicable for sheep.^7^ Quantification of bone area ratio followed the same procedure as histological analysis ("Animal model" section).

### Failure Risk Assessment

The failure risks of implant and periprosthetic bone were evaluated using maximum nodal von Mises stress (implant) and largest principal strain (bone), compared with their respective limits. Bone failure strain was assumed to be 0.0305 23 with strength and fatigue limits of titanium alloys given in Table 1. Custom-written post-processing scripts in Python were used to identify nodes with the highest von Mises stress and principal strain and vertical path plots across the nodes were drawn.

**Table 1:** Mechanical properties of Ti_6_Al_4_V, machined and additively manufactured using electron beam melting (EBM). Fatigue strength at 10^7^ cycles 21,32,34

	Machined	Additively manufactured
Yield strength (MPa)	903	882
Ultimate tensile strength (MPa)	958	979
Fatigue strength (MPa)	510	350

## Results

### Verification of FEA Model with *In Vivo* Data

**Figure 4:**
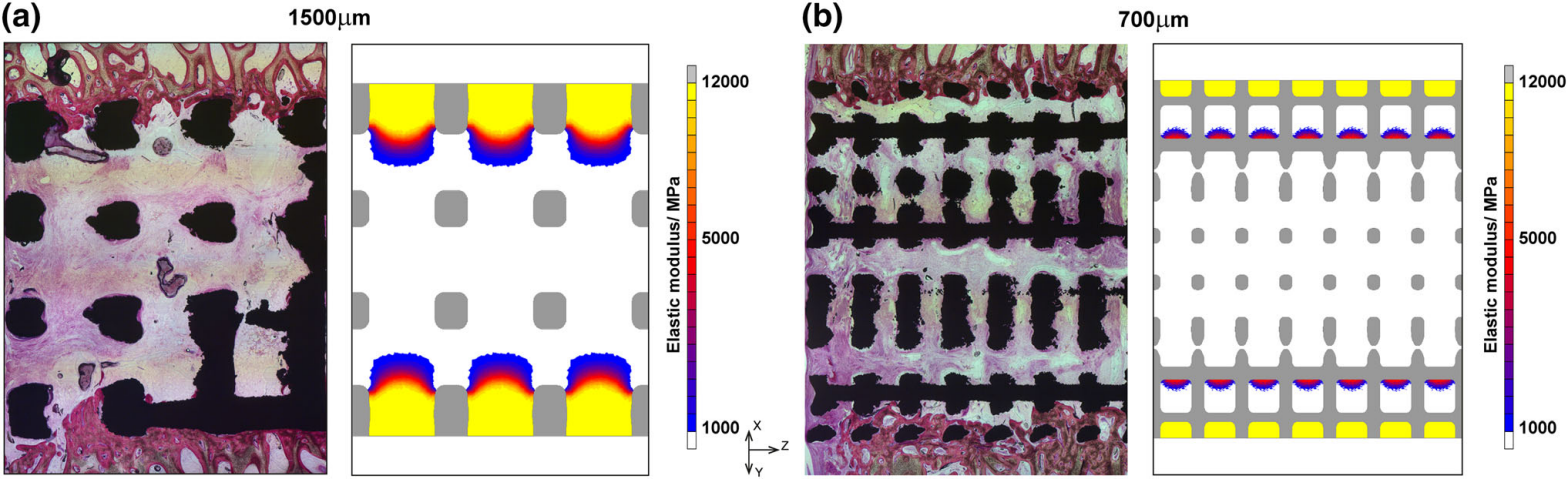
Correspondence of bone formation in animal model histology (left) and FEA models (right) for both implants obtained at equilibrium: (**a**) 1500 μm pore size model. (**b**) 700 μm pore size model; histology: bone stained pink with toluidine blue; FEA: scale shows elastic modulus (MPa).

**Figure 5:**
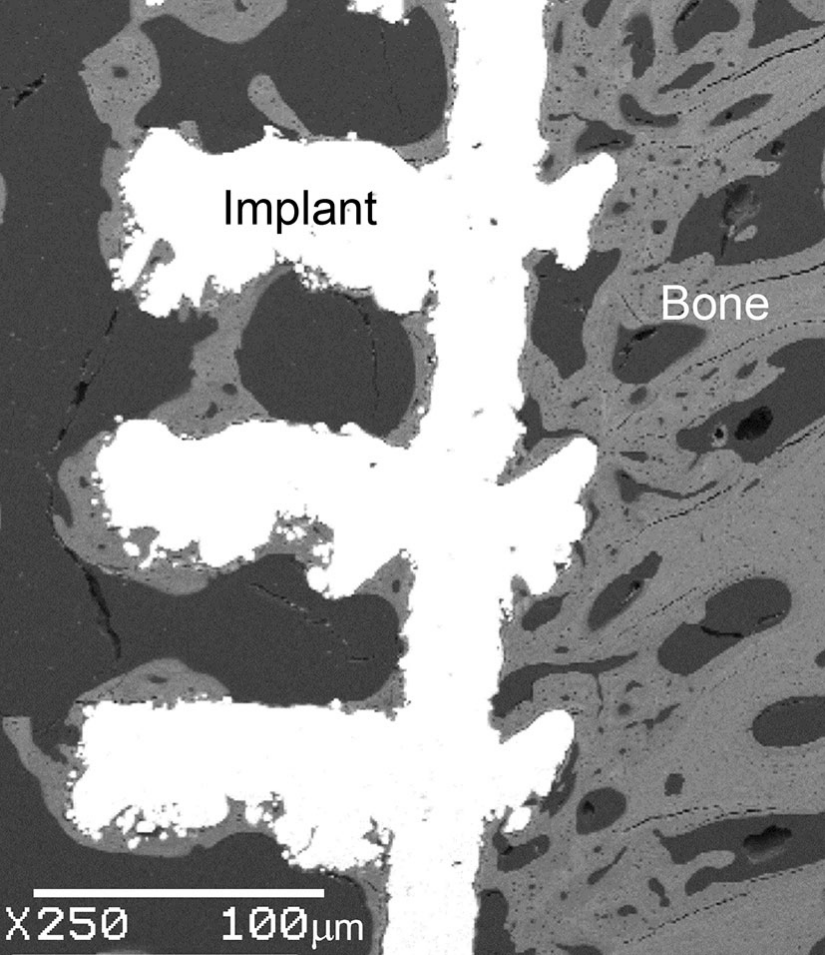
Back scattered scanning electron micrograph (SEM) of the bone-implant interface for a 700 μm implant, retrieved at 6 weeks after insertion.

Histology results showed regions of bone formation across the centre of longitudinal sections taken after 6 weeks (Fig. 4). No difference was observed between left/right leg, orientation (inside/outside) and location of the implants tested. A growth gradient was observed with the highest bone formation at the exterior, which reduced towards the centre. The percentage surface integration was 9.8 ± 5.0% and 10.7 ± 4.9% for the 1500 μm and 700 μm implant respectively, not significantly different (Mann–Whitney *U* test, *p* = 0.810). SEM images of bone formation within the pores showed that the bone is mature and lamellar in structure, indicating that the bone formed is unlikely to be a direct reaction to the surgery (Fig. 5). Regions of bone formation predicted by the FEA models at equilibrium qualitatively agreed with histology results as bone growth was restricted to the outer implant pores irrespective of pore size (Fig. 4). For the 1500 μm implant, numerical results predicted formation of dense bone in the outer pore, surrounded by the plates. For the 700 μm model, fully dense bone was predicted to form in the outer pore, with the strut blocking bone formation on its underside. There was bone formation originating from the top side of the second strut layer, to about 1/3 the height of the pore. For both implants, histology results showed bone formation only at the first strut and thus FEA models over-predicted bone growth. The bone surface ratio obtained from histomorphometric analysis of 6 implants (one from each sheep) were 0.125 ± 0.087 [range 0.041–0.288] and 0.134 ± 0.067 [range 0.021–0.222] for the 1500 μm and 700 μm implant respectively, not significantly different (Mann–Whitney *U* test, *p* = 0.575). Predicted percentage bone surface areas of the 1500 μm and 700 μm implants were higher than the histological results at 0.270 ± 0.052 [range 0.196–0.374] and 0.175 ± 0.047 [0.088–0.224] respectively, which were significantly different for the two implants (Mann–Whitney *U* test, *p* = 0.001).

### Changes to Implant Stress Distribution due to Bone Remodelling

**Figure 6:**
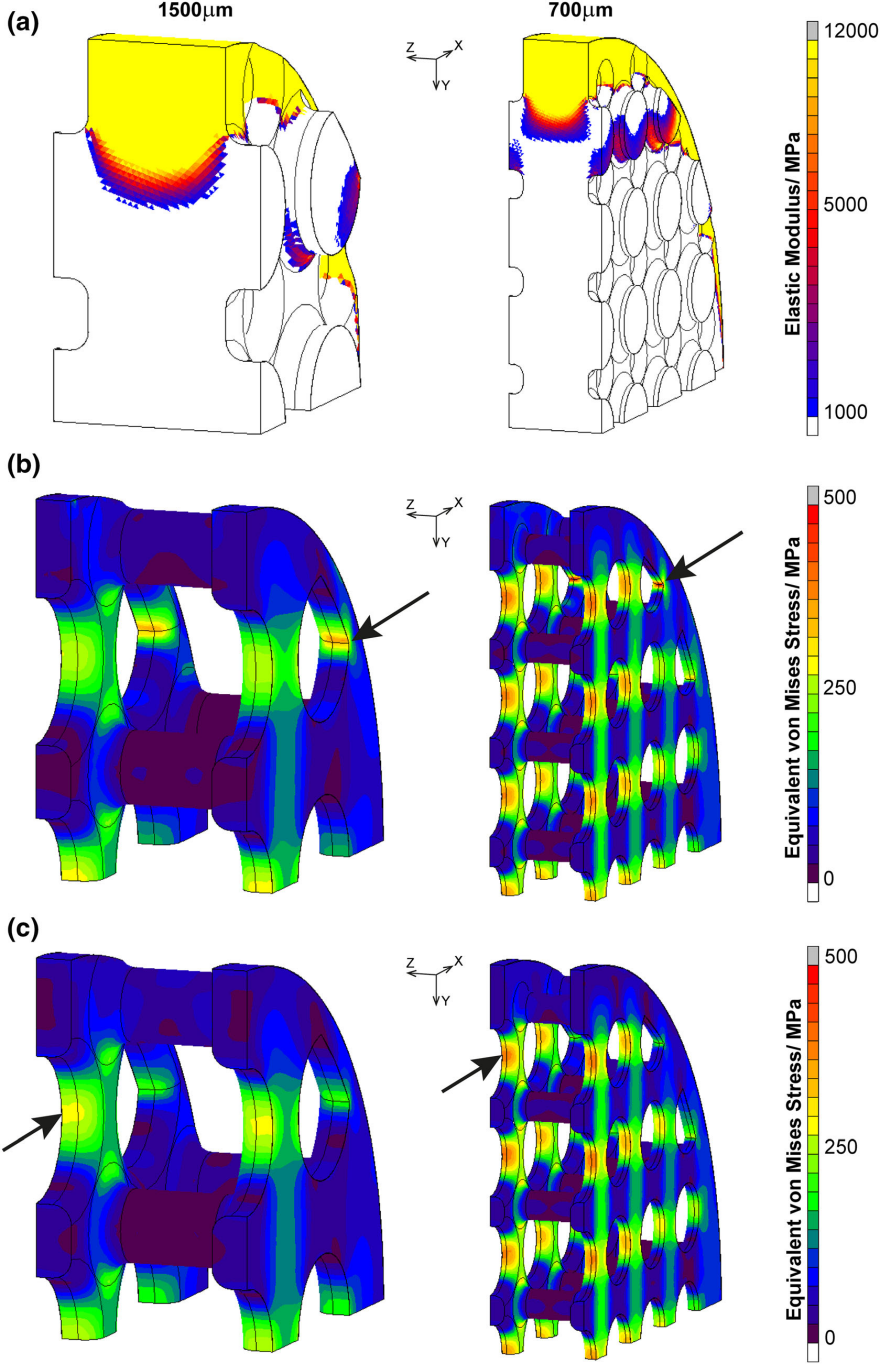
Effect of bone formation on von Mises stress in the implant. (**a**) Elastic modulus of tissues showing the extent of bone remodelling; Implant not shown for improved visualisation; (**b**) stress in implant before remodelling; (**c**) stress in implant after remodelling, at equilibrium; Arrows indicate regions of maximum von Mises stress.

Stress patterns in both porous implants changed due to bone remodelling. Before remodelling, the highest stress concentrations were at the sharp edges where partial holes intersect the outer ring of material (Fig. 6b). High stresses were also present along the plates, near holes along the loading direction, and at strut fillets. At equilibrium, bone formation extended beyond the top layer of struts in both implants and partially filled the first row of holes on the underside. The predicted depth of bone growth in the 1500 μm model was higher than in the 700 μm implant, which was not observed experimentally (Fig. 6a).

There was an overall stress reduction in both implants with remodelling and bone formation. The highest stress concentration in the 700 μm implant reduced by 23% from 534 to 411 MPa, and by 21% from 383 to 301 MPa for the 1500 μm implant (Table 2). Before remodelling, peak von Mises stresses of both implant models were lower than the tensile strength of machined Ti_6_Al_4_V (Table 1), but above the fatigue strength of machined Ti_6_Al_4_V at 10^7^ cycles for the 700 μm implant. The maximum von Mises stress exceeded the fatigue strength of untreated additively manufactured Ti_6_Al_4_V for both pore sizes (Tables 1, 2).

**Table 2:** Peak von Mises stress experienced by the implant before and after remodelling

Implant size (μm)	Peak von Mises stress (MPa)	
	Before remodelling	At equilibrium
1500	383	301
700	534	411

**Figure 7:**
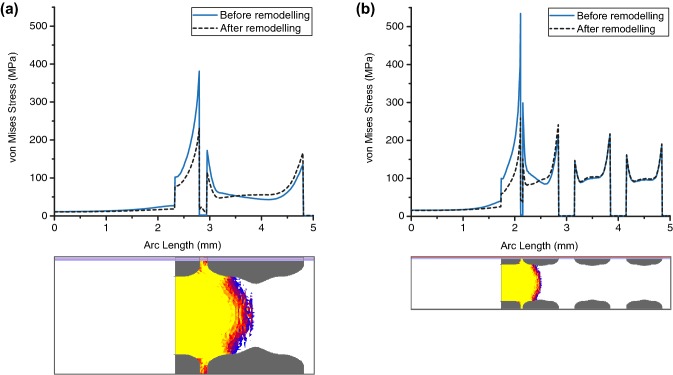
Stress plots along loading direction of the implant before and after remodelling (path across location of initial maximum nodal value). Cutting planes show extent of remodelling at equilibrium. (**a**) 1500 μm implant; (**b**) 700 μm implant.

Remodelling changed the location of the highest stress concentration, initially at the edges of irregularly shaped outer holes, to the holes along the plates after remodelling (Fig. 6b/c arrows). This reduction corresponded to regions with bone remodelling, primarily at the geometrical shape change, at the top of the plate and around the fillet above the struts. Stress reduction was observed in the implant adjacent to regions of remodelled bone, but there was a slight increase in stress inside the partially remodelled bone (Fig. 7). There was no observed change in stress levels inside the deeper struts, beyond an arc length of 3 mm for the 700 μm implant.

**Figure 8:**
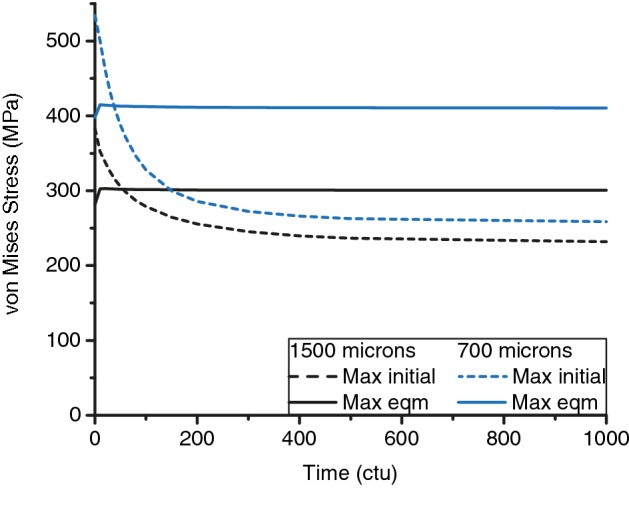
Time evolution of von Mises stress for 1500 μm and 700 μm implants at locations of highest initial stress (Max initial, at partial hole, Fig. 6b arrows) and highest stress at equilibrium (Max eqm, thinnest strut cross-section. Figure 6c arrows).

As adjacent tissues remodelled, the high stresses at the partial holes (Fig. 6b arrows) decreased asymptotically with time. Most of the reduction occurred within 100 ctu (Fig. 8 dashed lines). At equilibrium, von Mises stresses of the 700 μm and 1500 μm implants at the partial holes reduced to 259 MPa and 232 MPa, below the fatigue failure strength of additively manufactured Ti_6_Al_4_V. At equilibrium, peak stresses were located at the middle of the first layer of holes along the plates, the thinnest cross-section (Fig. 6c arrows). Stresses at these holes increased slightly from 282 to 301 MPa, and 398 to 411 MPa in the 1500 μm and 700 μm implant respectively (Fig. 8 solid lines). The increase in stress was due to remodelling that occurred above the holes, increasing the stress uptake in the local region that had not remodelled (Fig. 7). The maximum stress in the 700 μm implant at equilibrium was 37% higher than that in 1500 μm model, as the 700 μm implant has a higher porosity (75%) compared to the 1500 μm model (70%) and thus thinner struts and plates.

### Strain Within Remodelled Tissue and Bone

**Figure 9:**
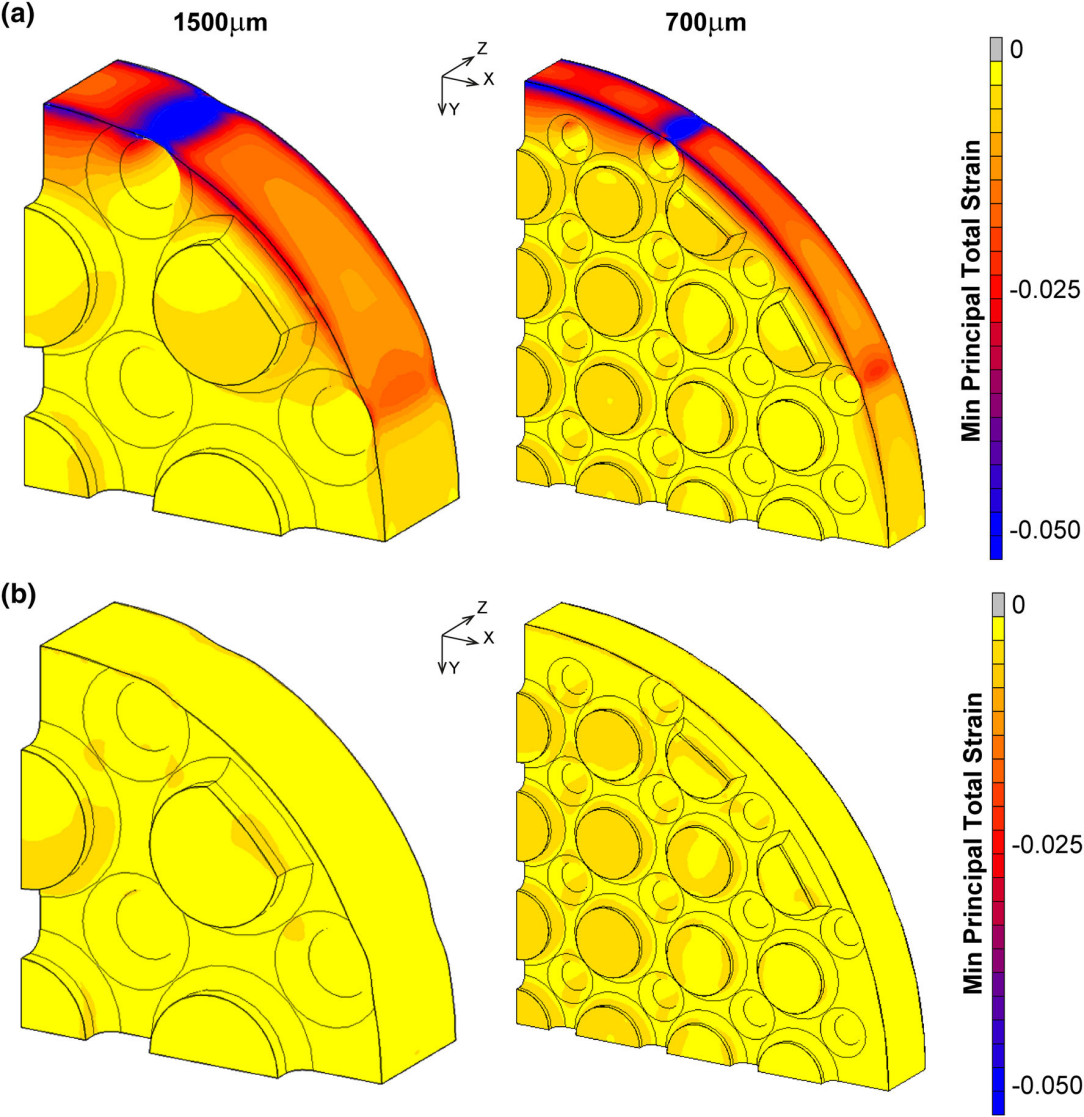
Minimum principal strain in tissue filling porous implant (left: 1500 μm pore size; right: 700 μm pore size). (**a**) Before remodelling. (**b**) At equilibrium after remodelling.

The highest absolute strains (minimum principal strain) before remodelling were at the circumference of the soft tissue in the loading direction, especially adjacent to the outer implant struts (Fig. 9a). The highest compressive strain for the 700 μm implant was 0.08, 10% higher than for the 1500 μm implant. Bone remodelling significantly reduced the compressive strain for both cases to less than 0.02 (Fig. 9b). For the 700 μm implant, there was a redistribution of the minimum principal strain in the tissues within the inner pores, with an increase in magnitude adjacent to the partial holes.

The peak strain magnitude in the bone stock before remodelling was 0.03, close to the fracture limit of ovine bone. Remodelling reduced the peak maximum principal strain to less than 0.008. The highest maximum principal strain remained towards the middle of the arc at 45°, but strain was redistributed across the full thickness of the bone stock, causing an increase in strain in the newly formed bone in the region between 30° and 60° from the vertical direction. However, these strains were not conducive to further bone formation deeper into the implant as the SED was below the threshold required for densification.

## Discussion

Extensively porous implants are being used in orthopaedics with considerable success.^3^,^22^,^35^ However, higher failure rates than expected have been reported 5 for the Tritanium acetabular component. Hence, changes in loading on the implant, tissue and bone, as bone grows into the porous structure, have important implications for the design of extensively porous implants. This study used a SED-based algorithm, developed to model extracortical bone formation,^7^ to evaluate the stress and strain distribution within porous implants of two different pore sizes (700 μm and 1500 μm) due to partial bone formation. Porous Implants made by SLS are being used clinically for applications to augment bone growth throughout the structure. The implant modulus depends on pore size and strut thickness, whilst the amount of bone ingrowth depends on implant modulus and loading conditions. The reliance of bone ingrowth on the modulus of a porous material has been demonstrated,^8^,^24^ and from this study it is apparent that modulus mismatch between the implant and the surrounding bone may lead to regions of the porous structure without bone tissue. The novelty of our study is that we predict bone ingrowth and relate this to the fatigue performance of substantially porous implants. We believe that this is important as short-term failures of these structure have been identified.^6^ In our study there was no bone formation in the centre of the implant, and overall stress in the implants reduced up to the depth where bone formation occurred (Fig. 7). There was a corresponding reduction in minimum principal strain in the surrounding bone stock and an increased stress uptake in the newly formed bone, as its elastic modulus increases. This agreed with previous *in vitro* and FEA studies that demonstrated that bone formation within porous implants improves the implant strength, yield stress and fatigue resistance, or reduces the risk of fracture through stress redistribution.^5^,^12^,^14^ However, the papers that investigated metallic implants considered homogenous bone at discrete stages of growth, whereas histology results from *in vivo* studies showed that bone formation is inhomogeneous, as porous structures are incompletely filled.^10^,^20^,^22^

The results showed that the highest initial implant stress concentration was located at irregular geometrical features, and the stress pattern in the plates in Fig. 6 was due to the narrowing of the cross section. The initial peak von Mises stress reduced quickly (within 100 ctu), and by more than 20% in both implant designs, as bone grew into the implant. This reduction is especially important for the 700 μm implant, as the initial stress exceeded the fatigue strength of Ti_6_Al_4_V. However, the predicted equilibrium von Mises stresses in the FEA models, 301 MPa and 411 MPa for the 1500 μm and 700 μm implants respectively, are still not within safe limits of untreated additively manufactured Ti_6_Al_4_V (fatigue strength: 350 MPa). As the peak stress at equilibrium was located at the holes, the results indicate that geometrical shape changes along the loading direction should be minimised where possible.

The as-designed geometries were used, but there could be geometrical discrepancies and worse fatigue performance due to current resolution limits of additive manufacturing.^2^,^14^,^18^ Titanium alloy is a notch sensitive material and both SLS and electron beam machining (EBM) lead to rougher surface finishing than conventional machining due to partially fused particles 27 that might increase stress concentration and lead to premature failure. It is difficult for surface finishing techniques to access the inner pores, and therefore the fatigue properties of these structures are likely lower than expected. Post-processing of titanium alloys through hot isostatic pressing (HIP) or heat treatment causes titanium alloy phase transformation, resulting in an increase in *β*-phase titanium that can increase the fatigue strength of Ti_6_Al_4_V by up to 40%.^15^,^27^,^34^ SLS and EBM may also lead to structures with lower strength due to increased porosity, hence HIP or other heat treatment is advised for alloy structures made using 3D printing. In this study, the porous structures were modelled as smooth, probably overestimating the fatigue strength. Future work should include conducting micro-CT scans of manufactured implants with metal artefact suppression, and using the reconstructed scans to improve the geometrical fidelity of the FEA models.^37^ The FE models did not take into account surface microgeometry or changes in the structure of the alloy such as grain size or phase changes. We expect that these changes would only reduce the fatigue strength of the porous material.

Bone remodelling was highest at regions of highest curvature, which agreed with the result obtained using a mechanobiological algorithm to model bone ingrowth.^3^ One of the goals in porous implant design is to induce bone formation throughout the entire implant, but this remains a challenge in orthopaedics as implant design is often conducted empirically, or to match the average surrounding bone mechanical properties.^2^,^14^,^18^,^24^,^33^ Path plots taken across the FEA models indicate that lack of internal bone formation seen *in vivo* is the result of stress shielding by the implant structure, reducing SED in the tissue to less than 3 MPa (Fig. 7). Thus, the effectiveness of partial bone formation in reducing stress concentration in the implant depends on the location and depth of bone remodelling. Therefore, a trade-off needs to be considered, as sufficiently high stress and strain in the surrounding tissue is required to enhance bone formation, but increases the risk of implant fatigue failure in the absence of bone remodelling.

Bone remodelling improves the mechanical interlock, beneficial for improved load transfer to existing bone stock to prevent long-term resorption. These results highlight the importance of implant design to optimise bone formation. Bone remodelling can reduce stress to be lower than regions unaffected by bone remodelling (Fig. 8), suggesting that implant architecture and stiffness are important since they can affect adaptive changes.^2^,^18^,^26^ Previous work that investigated the effect of reducing the apparent modulus mismatch showed that increasing porosity often increases bone formation.^3^,^26^ In particular, bone formation of up to 57% has been reported when Octet truss lattice structures were implanted in canine diaphysis.^2^ Another approach is to use a different biocompatible, low modulus, high strength titanium alloy. FEA simulations for a condylar defect model predicted that the use of Titanium-Tantalum alloy (*E* = 67 GPa) instead of Ti_6_Al_4_V (*E* = 110 GPa) would increase the volume of bone formation from 34 to 65%.^8^ However, the actual value for *in vivo* studies may be lower as the model over-predicted the amount of bone formation and these alloys are currently not widely used for SLS.

Large strains were observed in the thin layer of soft tissue between the implant and bone, and at the bone-implant interfaces before remodelling (Fig. 9). This is partially due to the modelled tied contact conditions; the assumption of friction interfaces could be better suited to model the press-fitted implants. Tied contact conditions were used as osseointegration was assumed, as Ti_6_Al_4_V is biocompatible and previous histology result showed that bone adjacent to the implant had osseointegrated.^9^,^22^ The initial values of − 0.08 and 0.04 for minimum and maximum principal strain exceed the 0.03 magnitude limit for ovine bone fracture. After remodelling, these values reduced to safe levels of − 0.02 and 0.01 respectively.^23^ The results suggest that care needs to be taken in the design of implants (e.g. placement of struts) to avoid thin layers of soft tissue experiencing high strains.

Implants were modelled as quarter slices, not considering potential end effects of the cylindrical implant and its location within the condyle on the stress distribution, as histological results did not show statistically significant differences in bone formation across the implant or its orientation and location 8 (Fig. 4). Peak load in medial condyles was applied in the FEA, as full weight-bearing immediately post-implantation was allowed. A uniform distributed load was assumed based on the weight of the animals used, a common technique.^1^ These sources of inaccuracies could explain the over-prediction of bone area ratio in the FEA model. Conducting full gait analysis with instrumented implants would increase the model fidelity. However, the use of a constant peak load represents the worst-case scenario for failure analysis, and it is commonly accepted in literature that bone tissue adapts to the peak stimuli during tissue healing.^16^

An initial, homogenous value corresponding to the average elastic modulus of trabecular bone in sheep at 80 months was assumed. Parametric analyses had been conducted on the choice of initial modulus and time step, but inhomogeneous material properties may affect the extent of bone formed.^7^ A limitation of using the SED-based bone remodelling algorithm is that the correlation between simulation (ctu) and actual time has yet to be established for this animal model, and the rate of bone formation needs to be interpreted with care. The results from this study cannot be used to calibrate the parameters of the bone remodelling algorithm as bone formation was assessed at only one time point. The FEA algorithm was evaluated at equilibrium and at this point there was a maximum absolute difference of 14.5% between the predicated levels of bone formation and the *in vivo* result. SEM (Fig. 5) showed that the bone formed at 6 weeks was lamellar in structure and not woven, indicating that it was relatively mature, but the *in vivo* data may not be at equilibrium as only one time point was investigated and compared with the equilibrium FEA results. Other biological factors could have contributed to the slower rate of bone formation, which were not included in the bone remodelling algorithm.

Bone remodelling algorithms are based on phenomenological models, which benefit from being less sensitive to the boundary conditions as the stress/strain environment around implants could not be accurately determined for this study. Phenomenological models have shown good agreement in predicting changes of bone density in humans.^11^ The use of homogeneous soft tissue was a placeholder for bone remodelling to occur, called granulation tissue by several authors.^5^,^11^,^19^ As the focus of this paper is on the interaction of bone formation with the implant, rather than tissue differentiation into phenotypes, the term soft tissue was used, and only mechanical stimulus was considered. Simulations could be improved by incorporating bone ingrowth. Bone formation was handled implicitly in the remodelling algorithm through the assumption of sequential bone growth. However, bone ingrowth could be modelled explicitly, and the differentiation of cells to form osteoblasts could be considered to model osteoinduction, by considering deviatoric, hydrostatic stresses and fluid flow.^5^,^19^,^25^

The results indicate that reduced initial loading could minimise implant failure risks. Load levels need to be large enough to stimulate bone formation and increase the mechanical strength at the bone-implant site, as experimental evidence has suggested that limiting loading could slow rehabilitation.^13^ As patient weight differs, the rehabilitation loading scheme should be patient-specific, with a gradual increase in loading to minimise both healing time and risk of implant failure. This study employed histological analysis (which is destructive), an important requirement for progressing towards clinical trials, to quantify the extent of bone ingrowth. Future work should consider larger studies with multiple time points, the inclusion of both non-destructive evaluation and testing of implants to failure, imaged using micro-CT, to further validate the FEA models and evaluate implant performance. A clinical tool could be developed to monitor bone regeneration progress, representing the elastic modulus as a percentage change to the pre-operative value, and the implant stresses as the ratio to material strength.

The effect of partial, inhomogeneous bone formation into the pores of additively manufactured implants was studied. An FEA algorithm previously developed to model bone formation was used to predict the effect of bone remodelling on the stress in the bone-implant structure. Results showed that bone remodelling protects the implant, reducing maximum von Mises stress by more than 20%. The maximum implant stress is still not within safe fatigue limits of additively manufactured Ti_6_Al_4_V and further improvements to implant design are suggested. Initial rehabilitation should be carefully implemented to load the structure within safe limits, while providing sufficient stimuli for bone formation to occur and to protect against implant failure. The use of extensively porous implants in load bearing situations should proceed with caution as the implant fatigue performance will be determined by the level of bone growth. Regions with only partial bone formation in the porous structure due to inappropriate structural stiffness may be at risk of fatigue failure.
